# Temporal Patterns of Fever Onset as an Indicator of Etiology in Intracerebral Hemorrhage

**DOI:** 10.3390/neurolint18040068

**Published:** 2026-04-03

**Authors:** Felix Hess, Enayatullah Baki, Julian McGinnis, Tun Wiltgen, Hannah Scholz, Kathleen Bernkopf, Gerhard Schneider, Jan Kirschke, Dominik Sepp, Bernhard Hemmer, Silke Wunderlich, Mark Mühlau

**Affiliations:** 1 Department of Neurology, School of Medicine and Health, Technical University of Munich, 81675 Munich, Germany; 2 Department of Anesthesiology and Intensive Care Medicine, School of Medicine and Health, Technical University of Munich, 80333 Munich, Germany; 3 Department of Computer Science, School of Computation, Information and Technology, Technical University of Munich, 80333 Munich, Germany; 4 Department of Diagnostic and Interventional Neuroradiology, School of Medicine and Health, Technical University of Munich, 80333 Munich, Germany; 5 Munich Cluster for Systems Neurology (SyNergy), 81377 Munich, Germany

**Keywords:** intracerebral hemorrhage, fever, pneumonia, central fever, infection, outcome

## Abstract

Background: Fever occurs frequently in patients with intracerebral hemorrhage (ICH) and is associated with worse functional outcomes. Rapid identification of the fever’s cause is crucial for guiding diagnostics and treatment. Data on the distribution of different fever causes in ICH are limited, and the diagnostic value of the day of fever onset remains uncertain. This study aimed to assess the distribution of fever causes in a large cohort of ICH patients and to evaluate whether temporal patterns of fever onset are associated with its underlying cause in a clinically meaningful manner. Methods: This retrospective single-center study included 547 patients with spontaneous ICH. Fever was defined as a body temperature exceeding 38.3 °C for at least two consecutive days. Fever causes were evaluated by two blinded investigators and categorized as infectious, central, or other causes. Infectious fever causes were further specified. Results: Fever occurred in 213 patients (39%) and was associated with longer hospital and ICU stays (both *p* < 0.01) and poor functional outcome (odds ratio 2.0, 95% CI 1.1–3.6). The three most frequent fever etiologies (>90% of cases) were pneumonia, central fever, and catheter-associated infections (i.e., urethral tract infections, ventriculitis, and central line-associated bloodstream infections). Median onset day differed across etiologies (overall *p* < 0.001): central fever developed earliest (2 [IQR 1–3] days), followed by pneumonia (5 [IQR 4–7] days) and catheter-associated infections (8 [IQR 5–12] days). Conclusions: In ICH, the day of fever onset may provide a useful clue to its etiology and could support clinical decision-making, but prospective validation is needed.

## 1. Introduction

Fever is a frequent and clinically significant symptom in patients with intracerebral hemorrhage (ICH) [1,2,3]. It impairs recovery from acute brain injury by increasing cerebral metabolic demand, promoting excitotoxicity and oxidative stress, and disrupting the blood–brain barrier, all of which aggravate secondary neuronal damage and lesion expansion. Consequently, it is associated with longer hospital stays, higher mortality, and worse functional outcomes, making fever a major therapeutic concern in stroke medicine. In addition, fever often coincides with other markers of systemic stress, such as leukocytosis and hemodynamic instability, which can further complicate neurocritical care management and obscure the distinction between primary brain injury and secondary systemic complications [3,4,5,6,7].

Infections such as pneumonia and urinary tract infections—conditions to which stroke patients are especially vulnerable because of dysphagia, immobility, and frequent use of invasive devices—constitute the most frequent causes of fever in the acute phase of stroke [3]. Alternatively, fever may result from stroke-related injury to brain regions involved in thermoregulation, such as the hypothalamus or brainstem, leading to a non-infectious hyperthermia known as central fever, which has been shown to occur particularly often in patients with ICH [8,9,10]. Central fever is typically resistant to antibiotics, may fluctuate despite negative cultures and imaging, and often coexists with severe neurological deficits, making its recognition clinically challenging [8,11,12].

Consequently, further diagnostic workup and treatment decisions, including the initiation and duration of empirical antibiotic therapy, crucially depend on the underlying cause of fever. Therefore, prompt and dependable determination of the fever etiology is crucial in everyday clinical practice, both to avoid delays in treating occult infection and to limit unnecessary antimicrobial exposure. The day of fever onset has recently emerged as a promising differentiator between central and infectious fever, suggesting that temporal fever patterns after stroke may represent an underappreciated indicator of the underlying cause [10,12]. However, data on the distribution of different etiologies of fever in ICH and on the diagnostic value of temporal fever characteristics—beyond the distinction between central and infectious fever—remain scarce, as most prior studies have treated fever as a largely homogeneous phenomenon and have not systematically disentangled distinct etiologies or their temporal patterns within the same ICH population [8,9]. In routine practice, the etiologic work-up of fever after ICH relies on clinical examination, laboratory markers, microbiological testing, and imaging, yet these investigations may be delayed, inconclusive, or non-specific, particularly early after hemorrhage. Temporal information, such as the day of first fever onset, might therefore provide easily available clues to the likely etiology and could help prioritize diagnostic pathways and resource use in busy stroke units. Against this background, there is a clear need for pragmatic, bedside-available indicators that can complement conventional infection work-up in patients with ICH. Simple clinical variables that are routinely documented in every patient and do not require additional testing are particularly attractive in this setting, as they can be incorporated into decision-making even in resource-limited environments. Temporal characteristics of fever fulfil these criteria and are readily accessible to treating teams from the first days of hospitalization. Yet, despite their intuitive appeal, temporal patterns have rarely been evaluated systematically in relation to specific fever etiologies after ICH [3,8,13].

Therefore, this study aimed to investigate the frequency of fever, the distribution of its most common causes, and its impact on clinical outcomes in a large cohort of patients with ICH, with a particular focus on whether the timing of the first fever onset helps to differentiate between the major specific etiologies of fever in this population, namely central fever and the most frequent nosocomial infections such as pneumonia, UTI, ventriculitis, and catheter-related bloodstream infection (CLABSI).

## 2. Methods

This study was performed in line with the principles of the Declaration of Helsinki. Approval was granted by The Clinical Ethics Committee (CEC) of the TUM University Hospital rechts der Isar (2024-568-S-NP) [12].

### 2.1. Patient Selection

**Figure 1:**
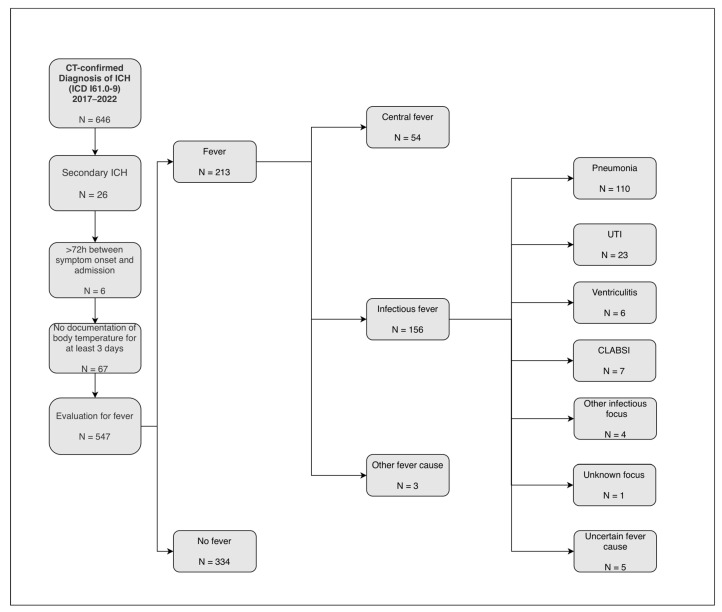
Flow diagram illustrating the patient selection process. Abbreviations: ICH = intracerebral hemorrhage, ICD = International Statistical Classification of Diseases and Related Health Problems, CT = computed tomography, UTI = urinary tract infection, CLABSI = central line-associated bloodstream infection, secondary ICH refers to ICH caused by vascular malformations, neoplasms, or hemorrhagic transformation of ischemic stroke.

In this retrospective single-center study, we included all patients admitted to our university hospital between January 2017 and December 2022 with a diagnosis of spontaneous intracerebral hemorrhage (ICH) coded according to the International Statistical Classification of Diseases and Related Health Problems (ICD-10 codes I61.0–I61.9) and confirmed by computed tomography. Patients were eligible if they were admitted within 72 h of symptom onset and had at least three documented body temperature measurements over a minimum of three consecutive days [12]. Patients with secondary ICH (i.e., ICH due to underlying vascular pathologies, hemorrhagic transformation of ischemic stroke, or brain tumors) were excluded. A flowchart of the patient selection process is provided in Figure 1. This resulting group largely coincides with the cohort of our recent imaging study on central fever in ICH [12].

### 2.2. Temperature Measurement and Fever Definition

Core temperature in our ICU and stroke unit is routinely measured using a bladder probe, which serves as the standard method. In patients without an indication for urinary catheterization—typically those with milder strokes who are continent and able to mobilize—temperature was instead assessed via the ear canal (17% of the cohort). Fever was defined as a body temperature exceeding 38.3 °C for at least two consecutive days, in line with previous studies on central fever in acute brain injury [10,12,14]. This threshold was chosen to focus on clinically relevant, sustained fever episodes and to minimize false-positive classification of brief, low-grade temperature elevations.

### 2.3. Determination of Fever Etiology

Fever was classified as infectious if there was either a clinical diagnosis of infection by the treating physicians, if the criteria for nosocomial infections of the Centers for Disease Control and Prevention (CDC), National Healthcare Safety Network (NHSN) [15] were fulfilled, or if there was culture growth of a pathogenic species [12]. Infection diagnoses were reevaluated using CDC/NHSN criteria (version 01/2025) by reviewing microbiological, laboratory, and radiological diagnostics, as well as progress notes.

Fever was classified as “other defined cause” if a specific non-infectious etiology was documented (e.g., rheumatologic disease, drug-induced fever, neutropenic fever).

Fever was classified as central if no infectious focus could be identified despite systematic review of clinical, laboratory, microbiological, and imaging data and if no other specific non-infectious cause of fever was documented [10,14]. Thus, central fever was defined as a non-infectious, neurogenic hyperthermia and established as a diagnosis of exclusion [10,14].

A trained investigator (H.S.) systematically collected data on body temperature measurements and potential causes of fever by reviewing medical records and daily progress notes from ICU and stroke unit patients. The investigator extracted information on infection diagnoses made by the treating physicians and on infection workup, including imaging reports (e.g., chest X-rays), urine studies, microbiological results, and laboratory parameters. Clinical signs of infection and other potential causes of fever, as documented by the treating team, were also recorded. In addition, we collected data on the day of fever onset relative to ICH symptom onset, the duration of fever episodes, and the use of antipyretic and antibiotic treatments.

After data collection, two investigators (F.H. and E.B.) categorized the type of fever (infectious, other cause, central, or uncertain) by reviewing each patient’s dataset while being blinded to potentially biasing information, such as the timing and duration of the fever episode, ICH characteristics, other patient characteristics, and functional outcomes.

The investigators initially had the option to rate the cause of fever as uncertain. Whenever the assignments differed or the cause was considered uncertain by either investigator, both investigators reached a consensus through discussion. If uncertainty remained after consensus discussion, the fever episode was classified as “fever of uncertain etiology” and was not assigned to any specific etiologic group. When a case was classified as infectious but could not be aligned with any infectious disease according to CDC/NHSN criteria, it was labeled as an infection with an unknown focus. In cases in which CDC/NHSN criteria were fulfilled for more than one nosocomial infection, the primary etiology of the first fever episode was defined by the infection first diagnosed by the treating team (7 cases in total; pneumonia in 6 patients and UTI in 1 patient), and subsequent infections were not considered in the present analysis. Inter-rater agreement for etiologic classification was evaluated using Cohen’s kappa.

### 2.4. Clinical Data

We collected data on demographic characteristics (including age and sex) and on cardiovascular risk factors such as hypertension, diabetes mellitus, atrial fibrillation, and prior use of antithrombotic medication. Stroke severity at admission was assessed by trained clinicians using the National Institutes of Health Stroke Scale (NIHSS). Functional outcome at discharge was determined with the modified Rankin Scale (mRS), and in-hospital mortality was recorded as an additional outcome measure. Furthermore, we documented total length of hospital stay and duration of intensive care unit (ICU) treatment for each patient.

### 2.5. Imaging Analysis

CT scans were visually assessed for lesion location (left vs. right hemisphere, deep vs. lobar, and infra- vs. supratentorial) and for the presence of accompanying intraventricular hemorrhage (IVH, graded as 0 for none or 1 for any). Parenchymal hemorrhage volumes were quantified using a 3D nn-UNet-based model for automated lesion segmentation, which represents the current gold standard for medical image segmentation and has demonstrated excellent accuracy in brain hemorrhage delineation [16,17]. Our nn-UNet model (Dice coefficient 0.92 in our validation cohort) has been previously validated and applied in earlier publications from our group [16,18]. In the present study, all automatically generated lesion masks were visually inspected and manually corrected when necessary to ensure precise hematoma delineation.

### 2.6. Statistical Analysis

Statistical analyses were conducted using IBM SPSS Statistics (Version 29.0.1). For descriptive comparisons, the dataset was divided into two groups (patients with at least one documented fever episode vs. patients without fever). Metric variables were compared using the *t* test, ordinal data with the Mann–Whitney U test, and binary categorical variables with the chi-square test; statistical significance was defined as a *p* value <0.05.

To assess the association between fever and functional outcome, we fitted a binomial logistic regression model with poor outcome (mRS 4–6 at discharge) versus good or moderate outcome (mRS 0–3) as the dependent variable. Candidate predictors were selected a priori based on previous work in intracerebral hemorrhage and clinical relevance and comprised age, NIHSS on admission, premorbid mRS, diabetes mellitus, oral anticoagulant use, infratentorial location, presence of IVH, PH volume, and fever status (any documented fever episode vs. no fever) [19,20]. Fever was thus treated as a binary exposure irrespective of its etiology or timing. From this full a priori model, we derived a parsimonious model by stepwise removing covariates with weak and non-significant associations while monitoring discrimination and calibration, thereby increasing the number of outcome events per variable, improving the stability of the regression estimates, and reducing the risk of overfitting. Model performance was evaluated in terms of both calibration and discrimination. For calibration, we report likelihood-ratio χ^2^ and several pseudo-R^2^ statistics (Nagelkerke, Cox–Snell, Tjur, and McFadden) derived from the fitted logistic regression model. Discrimination was assessed using the area under the receiver operating characteristic curve (AUC). Multicollinearity was assessed using tolerance values and variance inflation factors.

Finally, to compare the timing of fever onset between etiologies, we analyzed the onset day of the first fever episode across three major etiologic groups (central fever, pneumonia, and catheter-associated infections). Catheter-associated infections comprised urinary tract infection, CLABSI, and ventriculitis. We used a Kruskal–Wallis test, followed by prespecified pairwise post hoc comparisons with Holm-corrected *p* values, presenting corresponding medians, interquartile ranges, and pairwise statistics.

## 3. Results

Between 2017 and 2022, 646 patients with CT-confirmed ICH (ICD-10 I61.0–I61.9) were screened. Exclusions were applied sequentially: 26 patients had secondary ICH, 6 were admitted >72 h after symptom onset, and 67 had insufficient temperature documentation, leaving 547 patients for evaluation of fever, as shown in Figure 1. Patients had a mean age of 71.5 years, and fever occurred in 213 patients (39%). Overall, poor functional outcome (mRS 4–6 at discharge) occurred in 401 of 547 patients (73%), and in-hospital mortality occurred in 136 patients (25%).

### 3.1. Fever and Outcome

Compared with patients without fever, those with fever had higher NIHSS scores and larger hematoma volumes but were also younger and had better premorbid functional status, consistent with a profile of more severe acute brain injury in patients who were otherwise healthier at baseline (Table 1).

An association between younger age and the presence of fever was also found in the subgroup (N = 229) of patients with an infection diagnosis (without vs. with fever: mean age 78.0 ± 9.7 years vs. 67.3 ± 14.3, *p* < 0.001). In multivariable logistic regression, higher NIHSS on admission, older age, higher premorbid mRS, and the occurrence of fever were all associated with poor outcome, with fever remaining associated with poor outcome after adjustment (adjusted OR 2.00, 95% CI 1.1–3.6; *p* = 0.021; Table 2). Overall model fit was good (likelihood-ratio χ^2^ = 207.9, *p* < 0.001), and pseudo-R^2^ values indicated moderate-to-strong explanatory power (Nagelkerke R^2^ = 0.48; Cox–Snell R^2^ = 0.33; Tjur R^2^ = 0.39; McFadden R^2^ = 0.34). Discrimination was excellent, with an AUC of 0.88, indicating that the model accurately distinguished between patients with and without poor outcomes in most cases. The reduced model, including the five predictors NIHSS, Age, premorbid mRS, Fever (any vs. none), and PH volume, performed very similarly to the full a priori model, which additionally contained diabetes, oral anticoagulant use, infratentorial extension of ICH (yes vs. no), and presence of IVH, and is reported in Supplementary Table S1. Multicollinearity diagnostics showed tolerance values ranging from 0.8 to 0.9 and variance inflation factors between 1.1 and 1.3 for all included variables, indicating a low level of multicollinearity in our model.

### 3.2. Fever Etiology

Initial inter-rater agreement for etiologic classification of the first fever episode was >90%, with a Cohen’s kappa of 0.83 (95% CI 0.76–0.90), indicating almost perfect agreement.

**Figure 2:**
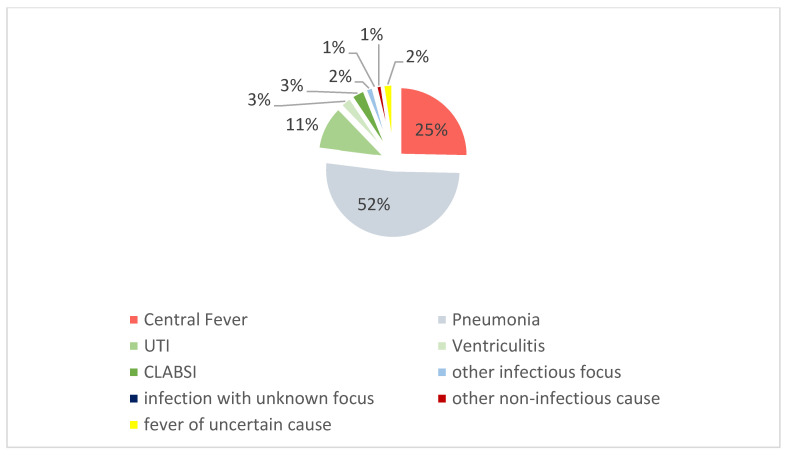
Frequencies of fever causes. The pie chart illustrates the distribution of fever causes among the 213 patients in the study cohort. Abbreviations: UTI = urinary tract infection, CLABSI = central line-associated bloodstream infection.

In 9.6% of cases, the cause was deemed uncertain by either investigator, or the categorization differed. After discussion, initial fever episodes in 5 patients remained uncertain or discrepant and were classified as fever of uncertain etiology (2%, Figure 2); they were not included in the etiologic subgroups used for onset-timing analyses.

Figure 2 shows the distribution of causes of first fever episodes. Pneumonia accounted for 52% of initial fever episodes, making it the most frequent cause. Central fever and catheter-associated infections contributed to 25% and 17%, respectively. Among the catheter-associated infections, urinary tract infections (UTIs) accounted for 11%, while ventriculitis and CLABSIs each contributed 3% to the causes of first fever episodes.

One case labeled as infectious did not meet the criteria for specific diagnoses and was categorized as an infection with an unknown focus. Additionally, three patients had their initial fever episodes due to other non-central or non-infectious causes (neutropenic, rheumatic, and drug-induced fever).

### 3.3. Association of Fever Onset and Etiology

**Figure 3:**
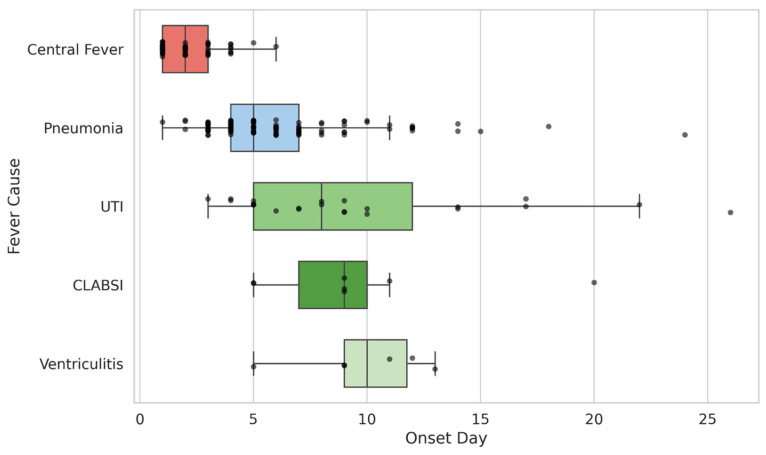
Fever causes by day of onset. The box plot displays the median, interquartile ranges and whiskers for the onset days of the most common fever causes in ICH. Abbreviations: UTI = urinary tract infection, CLABSI = central line-associated bloodstream infection.

As shown in Figure 3, central fever had the earliest onset, followed by pneumonia-associated fever (median onset on day 5), whereas fever due to catheter-associated infections (UTI, ventriculitis, CLABSI) occurred later, bearing in mind the smaller numbers in the CLABSI and ventriculitis subgroups. Consistently, Table 3 reports the Kruskal–Wallis test, demonstrating that onset days differed highly significantly between the three main etiologic groups, with all pairwise comparisons reaching statistical significance.

## 4. Discussion

Pneumonia, central fever, and catheter-associated infections were identified as the most frequent causes of fever in ICH, which was associated with poor functional outcomes, also in our cohort. The day of fever onset emerged as an indicator of its cause, which may not only help differentiate central from infectious fever but may also provide insights into the source of the infection. In everyday practice, such easily obtainable temporal information could support clinicians when deciding which diagnostic tests to prioritize and how aggressively to search for specific infectious foci in the acute and subacute phases after ICH.

As in previous studies, fever was associated with poor outcome even after adjustment for major baseline risk factors, although this observational design cannot exclude residual confounding by care intensity, infection burden, mechanical ventilation, dysphagia, or specific ICU interventions [1,4,8,10]. The frequency of fever in our cohort was 39%, which was comparable to studies on ICH using the 38.3 °C threshold [8,10]. However, the development of fever, especially as a symptom of infections, was age-dependent. The association between fever and a more favorable premorbid profile may partly reflect this age dependence of febrile responses, as younger patients are more likely to develop overt fever in the context of nosocomial infections, whereas elderly patients often show blunted temperature responses [21]. At the same time, this pattern is compatible with confounding by care intensity and survival profile, since younger, less disabled patients are more likely to receive prolonged invasive monitoring and survive long enough for infections and fever to become clinically apparent. Taken together, these observations support the view that fever in ICH represents both a biologically meaningful response and a surrogate of more complex clinical trajectories rather than a simple, isolated risk factor.

Studies that systematically investigated fever causes in patients with ICH are scarce [8,9].

These studies reported a higher proportion of non-infectious fever, which may be attributed to our inclusive definition of infections aimed at minimizing the risk of missing infectious cases [8,9]. However, pneumonia emerged as the most frequent cause of initial fever episodes in our cohort, which seems plausible given that it is the most common nosocomial infection in the acute phase of ICH; other studies have found frequencies of pneumonia similar to those in our cohort [2,22,23]. In contrast, UTIs were less frequently a cause of initial fever, with a febrile course of around 50%, whereas pneumonia went along with fever in approximately 75% of cases. Moreover, our focus on first (rather than subsequent) fever episodes may partly account for discrepant estimates of central versus infectious fever reported in previous studies.

We found a strong correlation between temporal patterns of fever onset and distinct causes of initial fever episodes. Besides central fever, which is known for its early onset [10], nosocomial infections also appear to exhibit characteristic timelines. Notably, our study specifically adds that there is a distinct temporal patterning of infectious fever in ICH patients. We hypothesize that during the first two days of ICH, central fever is the most common cause, as it can be seen as a symptom directly resulting from ICH-related disruption of thermoregulation [10]. In the subsequent days, pneumonia—most likely arising from aspiration, which occurs most frequently within the first 72 h after stroke [24], leading to febrile temperatures hours to days later—emerges as the predominant cause of first fever episodes, peaking around days 4 and 5 [2,23,25,26]. In contrast, UTIs, ventriculitis, and CLABSIs are catheter-associated infections that tend to occur later during treatment in the ICU or stroke unit [2,23]. These temporal patterns provide a pathophysiological rationale for using onset timing as an adjunctive clue and could, in future work, be translated into simple time-window-based diagnostic algorithms for fever after ICH.

We acknowledge the limitations of our study. First, its single-center and retrospective design requires validation in an external cohort, as local infection control protocols and patient population characteristics influence the time course of infections, potentially limiting the generalizability of these findings to other institutions. Second, a small proportion of episodes (2%) could not be assigned to a specific etiology even after consensus and were classified as fever of uncertain etiology, underscoring the inherent diagnostic uncertainty of retrospective adjudication. Third, our relatively restrictive fever definition (>38.3 °C for at least two consecutive days) and the mixed use of temperature measurement modalities (invasive bladder probes more often in severely ill patients vs. ear-canal measurements in milder strokes) may have led to underdetection or misclassification of brief or low-grade fevers and introduced some measurement bias. Fourth, the occurrence and timing of fever may have been influenced by prophylactic or empiric antibiotics and antipyretic treatment, which were not standardized and could have delayed or blunted febrile responses. Finally, our etiologic analyses were restricted to the first fever episode, and, in patients with multiple potential infectious sources, we assigned the primary etiology to the infection diagnosed first; together, these choices may underestimate the contribution of later or multicausal infectious episodes (including ventriculitis and catheter-related bloodstream infections) to the overall fever burden. Future prospective, multicenter studies with predefined fever assessment protocols will be essential to address these limitations and to confirm whether the temporal patterns observed here hold across different healthcare settings.

## 5. Conclusions

Fever onset day—an easily assessed parameter—may serve as an underrecognized indicator of its cause in acute ICH and warrants evaluation in clinical routine. Our findings suggest that timing of the first fever episode provides hypothesis-generating clues about the likely cause but does not constitute a validated triage tool, particularly given the retrospective, single-center design and residual uncertainty of etiologic adjudication. Thus, onset timing should be used only as an adjunctive signal within an integrated bedside assessment when prioritizing diagnostic work-up and treatment. In the longer term, incorporating temporal fever patterns into standardized fever management algorithms may help clinicians tailor diagnostic testing and antimicrobial strategies more efficiently. Prospective multicenter studies with predefined fever protocols are needed to validate these associations, refine clinically useful cut-offs for onset timing, and determine whether time-aware fever management can improve outcomes after ICH.

## Figures and Tables

**Table 1:** Overview of clinical, imaging and outcome parameters of patients with and without fever.

	No Fever N = 334	Fever N = 213	*p* Value
Clinical parameters			
Age, y (mean, SD)	74 (±13)	68 (±15)	<0.001
NIHSS (median IQR)	10 (5–16)	20 (14–30)	<0.001
Premorbid mRS (median IQR)	1 (0–2)	0 (0–1)	0.003
Use of Anticoagulants (%)	73 (22)	49 (23)	0.72
Diabetes (%)	68 (20)	40 (19)	0.68
Hypertension (%)	252 (75)	163 (77)	0.70
Imaging parameters			
PH volume (mean, SD)	36 (±42)	39 (±37)	0.38
Presence of IVH (%)	168 (50)	153 (72)	<0.001
Deep brain ICH (%)	108 (32)	104 (42)	0.22
Infratentorial ICH (%)	57 (17)	40 (19)	0.57
Hemisphere, left (%)	162 (49)	122 (57)	0.046
Outcome parameters			
Length of Hospital stay, d (median IQR)	12 (7–15)	22 (12–29)	<0.001
Length of ICU stay, d (median IQR)	2 (0–3)	11 (0–17)	<0.001
Poor outcome (mRS 4–6) (%)	213 (64)	188 (88)	<0.001
Mortality (%)	65 (20)	71 (33)	<0.001

Abbreviations: SD = standard deviation, IQR = interquartile range, NIHSS = National Institutes of Health Stroke Scale, mRS = modified Rankin Scale, ICH = intracerebral hemorrhage, PH = parenchymal hemorrhage, IVH = intraventricular hemorrhage, ICU = intensive care unit.

**Table 2:** Reduced multivariable logistic regression model for poor outcome (mRS 4–6 at discharge, coded as 1).

Predictor	Estimate (β)	Standard Error	Odds Ratio	95% CI for OR	*p* Value
NIHSS on admission	0.176	0.022	1.19	1.14–1.24	<0.001
Age (per year)	0.038	0.010	1.04	1.02–1.06	<0.001
Premorbid mRS	0.441	0.135	1.56	1.19–2.03	0.001
PH volume (per ml)	0.005	0.004	1.01	1.00–1.01	0.221
Fever (any vs. none)	0.692	0.300	2.00	1.11–3.60	0.021

Abbreviations: NIHSS = National Institutes of Health Stroke Scale, mRS = modified Rankin Scale, PH = parenchymal hemorrhage.

**Table 3:** Onset day of first fever episode by etiology.

Fever Etiology	n	Median Onset Day (IQR)	Comparison	z	*p* (Holm-Corr.)
Central fever (group 1)	54	2.0 (1.0–2.0)	1 vs. 2	−7.967	<0.001
Pneumonia (group 2)	110	5.0 (4.0–7.0)	2 vs. 3	−3.579 *	0.001 *
Catheter-associated infection (group 3) *	36	9.0 (5.0–11.3)	3 vs. 1	−9.347 *	<0.001 *

* Catheter-associated infection includes urinary tract infection, catheter-associated bloodstream infection, and ventriculitis. Groups: 1 = central fever, 2 = pneumonia, 3 = catheter-associated infection. *p*-values are Holm-adjusted post hoc pairwise comparisons after Kruskal–Wallis test.

## Data Availability

The data presented in this study are available on request from the corresponding author. The data are not publicly available due to ethical restrictions.

## Supplementary Materials

The following supporting information can be downloaded at: https://www.mdpi.com/article/10.3390/neurolint18040068/s1, Table S1: Multivariable logistic regression model for poor outcome (mRS 4–6 at discharge, coded as 1); AUC = 0.88, Fadden’s R2 = 0.345.
