# Supplementary material for: Temporal Patterns of Fever Onset as an Indicator of Etiology in Intracerebral Hemorrhage
Source: Neurol Int. 2026 Apr 3;18(4):68. doi: 10.3390/neurolint18040068 (PMC13119264; doi:10.3390/neurolint18040068)
Supplement: Supplementary file 1 [file neurolint-18-00068-s001.zip › neurolint-4116759-supplementary.pdf]

Supplementary Material

Supplementary Table S1. Multivariable logistic regression model for poor outcome (mRS 4–6 at discharge, coded as 1); AUC = 0.88, Fadden's R^2 = 0.345.

	Estimate	Odds ratio	95% Confidence Interval		P value
Age	0.035	1.036	1.015	1.057	**< .001**
NIHSS	0.175	1.192	1.141	1.245	**< .001**
mRS premorbid	0.442	1.558	1.190	2.035	**0.001**
Diabetes	-0.236	0.790	0.424	1.473	0.459
Use of anticoagulants	0.185	1.203	0.708	2.045	0.494
Fever	0.674	1.936	1.067	3.609	**0.030**
Infratentorial ICH	0.354	1.425	0.730	2.782	0.299
Presence of IVH	0.076	1.079	0.642	1.816	0.773
PH volume	0.005	1.005	0.997	1.013	0.205

Abbreviations: NIHSS = National Institutes of Health Stroke Scale, mRS = modified Rankin Scale, ICH = intracerebral hemorrhage, PH = parenchymal hemorrhage, IVH = intraventricular hemorrhage
